# Adipose Tissue Metabolic Function and Dysfunction: Impact of Burn Injury

**DOI:** 10.3389/fcell.2020.599576

**Published:** 2020-10-28

**Authors:** Supreet Kaur, Christopher Auger, Marc G. Jeschke

**Affiliations:** ^1^Ross Tilley Burn Centre, Sunnybrook Health Sciences Centre, Toronto, ON, Canada; ^2^Departments of Surgery and Immunology, University of Toronto, Toronto, ON, Canada

**Keywords:** browning, lipolysis, burns, adipose tissue, metabolism

## Abstract

For decades, adipose tissue had been considered as merely a storage depot and cushion to protect organs against trauma and injury. However, in recent years, a number of impactful studies have pinpointed the adipose tissue as an endocrine organ mediating systemic dysfunction in not only metabolic disorders such as obesity, but also in the stages following traumatic events such as severe burns. For instance, thermal injury induces a chronic β-adrenergic response associated with drastic increases in adipose lipolysis, macrophage infiltration and IL-6 mediated browning of white adipose tissue (WAT). The downstream consequences of these physiological changes to adipose, such as hepatomegaly and muscle wasting, are only now coming to light and suggest that WAT is both a culprit in and initiator of metabolic disorders after burn injury. To that effect, the aim of this review is to chronicle and critically analyze the scientific advances made in the study of adipose tissue with regards to its role in orchestrating the hypermetabolic response and detrimental effects of burn injury. The topics covered include the magnitude of the lipolytic response following thermal trauma and how WAT browning and inflammation perpetuate this cycle as well as how WAT physiology impacts insulin resistance and hyperglycemia post-burn. To conclude, we discuss how these findings can be translated from bench to bedside in the form of therapeutic interventions which target physiological changes to WAT to restore systemic homeostasis following a severe burn.

## Introduction

Adipose tissue, primarily composed of adipocytes, preadipocytes, fibroblasts, leucocytes, and endothelial cells, is widely recognized as a metabolic organ playing a central role in regulating whole-body energy homeostasis (Ahima and Flier, 2000; Coelho et al., 2013). Adipose tissue stores energy in the form of lipids and modulates their systemic mobilization and distribution in the blood (Sethi and Vidal-Puig, 2007). The incoming surplus energy from food is stored in adipocytes in the form of lipids or tri-acylglycerides (TAG) through the lipogenesis pathway (Tan and Vidal-Puig, 2008). Evolutionarily, adipose tissue functions to store incoming fat supply in the existing adipocytes by increasing the adipocyte size (hypertrophy) or by expanding the number of new adipocytes by adipogenesis (hyperplasia) (Ghaben and Scherer, 2019). However, prolonged reduced energy expenditure results in the expansion of adipocytes and subsequently obesity and associated metabolic syndrome (Lafontan and Langin, 2009). Hence, when lipid influx exceeds the adipose tissue storage capacity, the lipids start accumulating in ectopic tissues resulting in metabolic dysfunction and insulin resistance due to lipotoxicity (Virtue and Vidal-Puig, 2010). Several recent reviews have detailed the role of adipose tissue in preventing lipotoxicity and metabolic dysfunction in healthy versus obese individuals (Boura-Halfon and Zick, 2009; Virtue and Vidal-Puig, 2010; Nielsen et al., 2014; Fasshauer and Bluher, 2015; Zhu and Scherer, 2018; Ghaben and Scherer, 2019). Besides, it is now clear that adipose tissue not only serves as an energy reservoir but also acts as an endocrine organ and secretes metabolites and other signaling components such as adiponectin, leptin, and certain growth factors that play an essential role in multiple signaling pathways (Fasshauer and Bluher, 2015). The metabolites secreted by adipose tissue are commonly referred to as “adipokines.” Other examples of adipokines include visfatin, resistin, chemerin, apelin, interleukin 6 (IL6), interleukin 1β (IL1β), tumor necrosis factor α (TNFα), and fibroblast growth factor 21 (FGF21) (Fasshauer and Bluher, 2015). Some key examples illustrating the role of adipokines in coordinating metabolic function include (but are not limited to): hepatic glucose output (adiponectin, FGF21), insulin sensitivity (adiponectin, leptin), inflammation (IL6, IL1β, TNFα), and lipid metabolism (cluster differentiation factor 36) (Fasshauer and Bluher, 2015; Zhu and Scherer, 2018).

Adipose tissue is one of the largest endocrine organs in humans and is primarily of two types: white and brown. Both these adipose tissue types have distinct morphology, characteristics, and functional aspects. Brown adipose tissue (BAT) is specialized in heat production (or thermogenesis) and is commonly present in neonates and adults (Ahima and Flier, 2000; Coelho et al., 2013; Peirce et al., 2014). Initially, it was thought that BAT has no major physiological relevance in adult humans. However, recent studies using positron emission tomography – computer tomography (PET-CT) have revealed that enhanced BAT activity is positively correlated with resting energy expenditure. Also, PET-CT studies using the glucose analog 2-fluoro-2-deoxy-D-[^18^F]glucose (^18^FDG) have revealed the presence of focal areas of enhanced tracer accumulation, in the supraclavicular region, peri-adrenal region, axilla, intercoastal region, and around the great vessels in adult humans (Carter et al., 2011a,b). BAT, in comparison to white adipose tissue (WAT), has enhanced mitochondrial content, increased expression of uncoupling protein 1 (UCP1) and is more vascularized, allowing it to produce heat via fatty acid oxidation which results in smaller size multilocular adipocytes (Peirce et al., 2014). Although BAT stores lipids as well, its main function is heat production rather than nutrient supply.

**FIGURE 1:**
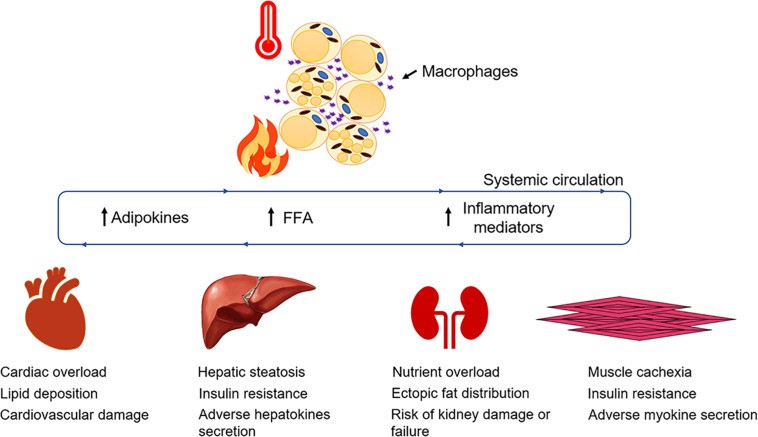
Adipose dysfunction after burn injury. WAT undergoes lipolysis and browning mediated by several signaling mediators and adipokines that contribute to enhancing systemic FFA flux and resting energy expenditure.

White adipose tissue, on the other hand, has larger unilocular adipocytes, reduced mitochondrial content, and lacks UCP1 expression. WAT is dispersed throughout the body and is mainly of two types: subcutaneous (inguinal in rodents) and visceral (epididymal in rodents) adipose tissue. WAT acts as a metabolic organ and handles a variety of metabolic functions while maintaining energy homeostasis in humans (Bartelt and Heeren, 2014; Peirce et al., 2014), thus making it a lucrative therapeutic target against metabolic disorders such as type 2 diabetes and obesity. Physiological changes to adipose tissue currently pose a major clinical challenge in the treatment of hypermetabolism, a chronic metabolic disorder occurring in severely burned patients. Indeed, the catabolism of lean muscle and adipose which is associated with the hypermetabolic response is devastating and a major contributor to the morbidity and mortality of patients (Jeschke et al., 2011). Burn injury and its associated hypermetabolic response initiates a sequelae of metabolic stress events in patients, characterized by elevated levels of glucose, insulin, free fatty acids, amino acids, and stress hormones in the serum (Jeschke et al., 2011), which triggers vital organs such as the liver, pancreas, muscle, adipose tissue, heart, and brain, to function at an accelerated rate. Once activated, hypermetabolism results in a vicious cycle of feed-forward loops in burn patients, ultimately resulting in enhanced resting energy expenditure (REE), increased risk of sepsis, infections, and ultimately death (Jeschke et al., 2011; Auger et al., 2017). The prevalence of hypermetabolism among severely burned patients has sparked a great interest in understanding the pathophysiological mechanisms underlying this response in order to minimize the adverse metabolic consequences of hypermetabolism such as insulin resistance, dyslipidemia, hepatosteatosis, and cardiovascular complications (Figure 1). Although the role of adipose tissue in efficient caloric storage and protective shielding is seemingly clear, we and others have recently begun to understand the profound impact of adipocyte metabolic dysfunction in elevating the hypermetabolic response post burn injury. Indeed, the burn-induced shift from caloric storage to a catabolic state disrupts energy homeostasis and sets in motion the systemic lipotoxicity and associated lipid accumulation in vital organs such as the heart, liver, and kidneys, which contributes to organ dysfunction. An intriguing question remains: what are the factors triggering adipose tissue dysfunction post burn injury and how are they regulated? This review aims to critically analyze our understanding and scientific advances made thus far on the mechanistic role of adipose tissue and beneficial aspects of therapeutic targeting of adipose tissue lipolysis and browning in order to mitigate the hypermetabolic response in burn patients.

## Adipose Tissue Lipolysis

Adipose tissue enacts lipolytic pathways when activated by external stimulus from the brain and adrenal glands during starvation mode, while exercising and when energy requirements are enhanced (Nielsen et al., 2014). Once lipolysis is activated, TAG reserves in adipocytes are broken down to glycerol and free fatty acids (FFA) that can be used when glucose reserves are running low in the systemic circulation (Lafontan and Langin, 2009). The released glycerol and FFA enter the bloodstream and subsequently help fuel vital organ function and maintain systemic energy homeostasis. These released lipids can be metabolized by essential organs as a substrate for β-oxidation and adenosine triphosphate (ATP) production (Kolditz and Langin, 2010). This unique ability of adipose tissue to balance storage and usage of lipids enables individuals to have an increased FFA buffering capacity to meet energy demand (Frayn, 2002). However, the metabolic consequences of an excessive expansion in adipose tissue reserves result in obesity, adverse adipokine secretion, and associated metabolic dysfunction (Alberti et al., 2009).

In adipocytes, the breakdown of lipid droplets is achieved by sequential action of three lipases that breaks down TAG into three FFA. In mammalian lipolysis, TAG is converted to di-acylglyceride (DAG) by tri-glyceride lipase (ATGL), which is a rate-limited enzymatic step (Zimmermann et al., 2004). DAG is further converted to mono-acylglyceride (MAG) by the sequential action of hormone-sensitive lipase (HSL) that has substrate specificity toward DAG (Haemmerle et al., 2002). MAG is further cleaved by the action of mono-glyceride lipase (MGL) that converts it into glycerol and FFA (Fredrikson et al., 1986). The activation and deactivation of lipolytic pathways is tightly regulated in adipose tissue by hormonal and nutritional abundance in circulation. The positive regulator and activators of adipose tissue lipolysis are catecholamines, natriuretic peptides, and stress hormone action that act on β-adrenergic receptors. Conversely, anti-lipolytic action is mediated by insulin (Jensen and Nielsen, 2007), catecholamines and hormonal action on α-adrenergic receptors (Laugwitz et al., 1996; Cho et al., 2005; Endo and Kobayashi, 2012; Rodrigues et al., 2013). Studies in burn patients have shown the increased systemic lipid levels and marked increase in the levels of plasma catecholamines and stress hormones suggesting the interlinked role of stress hormones in triggering WAT lipolysis after burn injury (Wolfe et al., 1987a,b; Jeschke et al., 2008, 2011). Moreover, a study conducted in pediatric burn patients using propranolol (β-adrenergic blocker) or placebo showed reduced expression of adipose genes related to lipid metabolism in propranolol-treated patients (Barrow et al., 2006).

In physiological conditions, the lipolytic pathway is activated by binding of catecholamines to β-adrenergic (predominately by β1 and β2, and to some extent by β3 in humans) receptors (Barbe et al., 1996; Jocken and Blaak, 2008). Conversely, anti-lipolytic action is mediated by binding to α2 adrenergic receptors (Berlan and Lafontan, 1982; Lafontan and Berlan, 1993). Both the α and β adrenergic receptor groups belong to the G-protein coupled receptor family, where α2 associates with the proteins containing G_i_ inhibitor subunit and β receptor associates with the proteins containing G_s_ stimulating subunit, to interact with adenylyl cyclase (Barrow et al., 2006). Upon activation, G_i_ inhibits adenylyl cyclase while G_s_ activates adenylyl cyclase. In turn, adenylyl cyclase converts ATP into cytosolic adenosine monophosphate (cAMP) that results in rapid accumulation of intracellular cAMP levels, which activates protein kinase A (PKA) signaling (also known as cAMP-dependent signaling). This whole process facilitates the activation of ATGL and phosphorylation of HSL that ultimately triggers the adipose tissue lipolysis cascade. More details on the activation and signal transduction of PKA-signaling are detailed elsewhere (Nielsen et al., 2014).

Enhanced FFA and systemic lipid levels are commonly associated with severe burn injury and the hypermetabolic response (Jeschke et al., 2011). In fact, enhanced lipolysis can persist in severe burn-injured patients in a timeframe ranging from a few days to years after the initial injury, resulting in adipose tissue wasting and systemic lipotoxicity (Jeschke et al., 2011). Adipose tissue lipolysis after burn injury is stimulated by adrenergic stress signaling that involves catecholamines, glucocorticoids, and cytokines mediated by the hypermetabolic response. Catecholamines such as epinephrine and norepinephrine as well as stress hormones are primary mediators of the metabolic response in adipose tissue via adrenergic receptors (Robidoux et al., 2004). In burn patients, levels of epinephrine, norepinephrine and cortisol are found to be enhanced and this triggers various catabolic events such as lipolysis, muscle wasting and enhanced REE (Jeschke et al., 2011, 2015). For instance, one research study in pediatric burn patients demonstrated elevated levels of catecholamines for 2 years in comparison to healthy individuals (Kulp et al., 2010). In another study, norepinephrine levels were noted to be 10-fold higher in urine samples and remained elevated for more than a year (Jeschke et al., 2011).

## Insulin Resistance in Adipose Tissue

Adipocytes are sensitive to insulin action and halt lipolysis to maintain systemic energy homeostasis (Ghaben and Scherer, 2019). Insulin binds to the insulin receptor substrate (IRS) α subunit on the external cell surface leading to conformational changes in the β-subunit for binding of ATP. Binding of ATP triggers the auto-phosphorylation of IRS β-subunit inside the cell that activates tyrosine kinase activity and enables further cellular activity of insulin (Wolever, 1990; Burks and White, 2001; Kido et al., 2001). There are four known types of IRS (1–4) (Burks and White, 2001). IRS-3 is present only in adipose tissue. Phosphorylation of IRS protein by insulin activates a variety of signaling pathways. For instance, activation of the phosphoinositol-3-kinase (PI3K)/protein kinase B (AKT) pathway promotes *de novo* lipogenesis, inhibits WAT lipolysis, and suppresses hepatic gluconeogenesis (Burks and White, 2001). Intracellular transport of glucose is mediated by insulin action via glucose transporter 4 (GLUT4) to promote glucose uptake, lipogenesis and suppress lipolysis that reduces FFA flux in blood stream (Smith, 2002). Therefore, in an insulin deficient state, adipose tissue liberates FFA to maintain energy homeostasis by fatty acid oxidation and for direct utilization by organs such as the liver, heart, and muscle. In addition, the liver converts FFA to ketone bodies which is an alternative substrate for the brain during fasting periods (Wilcox, 2005).

Severe burn injury is often associated with systemic insulin resistance, hyperglycemia and hyperlipidemia that significantly contributes to the morbidity and mortality in burn patients (Jeschke et al., 2011, 2020). Therefore, insulin therapy and similar analogs are considered an important therapeutic measure against the hypermetabolic response and have shown promising results in critical burn patients (Jeschke et al., 2010, 2016). Under normal circumstances, a postprandial elevation in blood glucose levels stimulates the release of insulin from pancreatic β-cells. Released insulin thereby promotes glucose uptake in skeletal muscle and adipose tissue, and inhibits glycolysis, glycogenolysis and lipolysis to maintain euglycemia (Belfort et al., 2005; Gormsen et al., 2007; Kolka et al., 2015). In critical circumstances such as severe burn injuries, the release of stress mediators causes metabolic derangements and significantly affects energy homeostasis. To meet the enhanced energy demand in stress conditions, the stress mediators oppose the anabolic action of insulin (inhibiting glucose production) causing a state of insulin resistance. Catecholamines inhibit insulin action during stress or exercise through α2 adrenoreceptors (Wilcox, 2005), suggesting the role of adrenergic stress signaling in promoting the insulin resistance state. These hypermetabolic conditions activate alternative energy sources such as adipose tissue lipolysis, hepatic glucose release, and skeletal muscle proteolysis (Jeschke et al., 2020). Thus, hyperglycemia and insulin fail to inhibit lipolysis and glucose production. Enhanced systemic energy circulation results in lipotoxicity, insulin resistance, and associated ectopic fat deposition in vital organs, thus affecting their function. Therefore, adipose tissue lipolysis and insulin resistance plays an intriguing role in contributing to a burn-induced hypermetabolic state.

## Brown Adipose Tissue Thermogenesis

Brown adipose tissue activation implies triggering BAT to generate heat by “non-shivering thermogenesis.” BAT was initially thought to primarily involve heat generation in neonates and hibernating animals with little contribution in metabolism (Abdullahi and Jeschke, 2017). Recent studies using PET-CT scan have shown the metabolic activation of BAT in response to different stress stimulators such as cold exposure, burn injury, and cutaneous wound (Carter et al., 2011a,b). Intriguingly, mice subjected to burn injury had reduced lipid content, and enhanced UCP1 content in BAT (Carter et al., 2011a,b). Furthermore, Yo et al. (2013) also showed that BAT activation is positively correlated with increased energy expenditure, augmentation of mitochondrial biogenesis and enhanced *Ucp1* expression. Upon targeting mitochondrial activity using SS31, Yo et al. (2013) showed that the mitochondria targeted peptide SS31 resulted in decreased energy expenditure in burn mice and suppressed *Ucp1* expression in isolated BAT from burn mice. More recently, Bhattarai et al. (2020) have shown the time-dependent recruitment of BAT post-burn injury, where UCP1-dependent respiration is augmented in the first 24 h post-burn and decreases thereafter. Overall, studies assessing the role of BAT when challenged with burn injury have revealed that BAT activates afterward with evidence of enhanced *Ucp1* expression. However, the insight into the role of BAT when challenged with burn injury is very limited. Further studies are required to determine if this depot contributes to systemic dysfunction via enhanced lipolysis or inflammatory mediators.

## White Adipose Tissue Browning

The concept of WAT browning implies triggering WAT to transition into brown-like adipose tissue known as beige adipose tissue. The latter is characteristic of an intermediate state between WAT and BAT, having a mixture of unilocular and multilocular adipocytes, enhanced mitochondrial content and exhibiting UCP1 expression (Bartelt and Heeren, 2014; Peirce et al., 2014). Research shows that WAT and BAT originate from different mesenchymal stem cell lineages and have different myogenic signatures (Timmons et al., 2007; Seale et al., 2008). However, beige and white adipocytes share the same cell lineage and white adipocytes can transdifferentiate into beige adipocytes upon stimulation (Park et al., 2014; Ikeda et al., 2018). In theory, beige adipocytes can originate from progenitor cells residing within WAT in response to external stimuli (Wang et al., 2013). Alternatively, they can also transdifferentiate from WAT or BAT that involves direct conversion of WAT or BAT into beige adipocytes and vice versa (Barbatelli et al., 2010; Rosenwald et al., 2013). Both WAT and BAT are highly innervated and sensitive to energy demand transmitted by the sympathetic nervous system that regulates the transcriptional circuit of the browning process.

In response to cold exposure or catecholamines, WAT browning is initiated by peroxisome proliferator-activated receptor γ (*Ppar*γ) that further activates thermogenic gene (*Pgc1*α, *Prdm16*, and *Ucp1*) expression. In addition, WAT browning can be activated by several hormones via crosstalk between tissues. For instance, FGF21 and bone morphogenetic protein 4 (BMP4) that are produced in response to adrenergic signaling can promote WAT browning (Fisher et al., 2012; Grefhorst et al., 2015). Other WAT-released hormones such as leptin and insulin also promote WAT browning through proopiomelanocortin (POMC) neurons and the sympathetic nervous system (Dodd et al., 2015). Furthermore, although catecholamines are required for the activation of WAT browning, M2 macrophages resident in WAT are also a source of catecholamines (Nguyen et al., 2011; Abdullahi et al., 2019a), suggesting a feed-forward loop of initiating WAT browning and its associated contribution to enhancing REE and WAT lipolysis. Overall, WAT browning can be triggered by energy sensing, metabolic demand, hormonal activation and adrenergic signaling, all of which suggest a crucial metabolic role for WAT in energy homeostasis.

Evolutionarily, WAT acts as an excellent thermal insulator that maintains body temperature and energy balance in humans. Also, activating WAT browning can potentially help obese or overweight individuals aiming to reduce body weight and become metabolically healthy (Bartelt and Heeren, 2014). However, stress conditions (such as severe burn injury) also trigger WAT browning that results in enhanced REE, enhanced mitochondrial content, UCP1 expression and abundant nutrient supply by triggering WAT lipolysis for fatty acid oxidation. WAT browning is seemingly a common attribute in severe burn-injured patients and is a major factor that fuels the hypermetabolic response in critically injured patients (Abdullahi and Jeschke, 2017). Therefore, WAT browning can be perceived as a dysfunctional trait in adipose and novel challenge for clinicians to deal with while treating severe burn-injured patients. Although significant success has been achieved in identifying the critical precursors triggering WAT browning, research is ongoing to identify measures to inhibit WAT lipolysis and browning in severe burn-injured patients (Abdullahi and Jeschke, 2017). In fact, a study conducted in pediatric and adult burn patients has demonstrated that WAT browning is a major driver of the hypermetabolic response and associated metabolic dysfunction (Patsouris et al., 2015; Sidossis et al., 2015). Furthermore, rodent studies have revealed the critical role of cytokines such as IL6 and macrophages in activating WAT browning and causing hepatic steatosis post burn injury (Abdullahi et al., 2017, 2019a).

## Adipose Tissue Inflammation – A Vicious Cycle

Inflammation in an individual’s cells or tissue is a multi-level response mechanism for communication and protection from any harmful substance and to prepare a protective shield to fight against it. In most of the cases, inflammatory triggers vanish as soon as the problem is resolved. However, in some cases, when the trigger is constant, the ongoing acute inflammation can turn into chronic inflammation (Barrett et al., 2019). In stressed conditions, adipocytes can actively produce and recruit other inflammatory cells and mediators that are capable of activating and recruiting immune cells such as macrophages and T-lymphocytes (T-cells) (Abdullahi et al., 2019a). WAT acts as a reservoir from which a myriad of metabolic signals can originate via the secretion of a plethora of cytokines and hormones. For instance, TNFα can directly disrupt insulin signaling (Hotamisligil, 1999) and IL6 can activate WAT browning (Abdullahi et al., 2017), leading to the development of insulin resistance and enhanced REE, respectively. Severe burn-injured patients often experience chronic inflammation and associated metabolic dysfunction (Jeschke et al., 2011). Such chronic hyper-inflammation often affects wound healing, triggers WAT browning, lipolysis, lipotoxicity, sepsis, and associated multi-organ failure complications.

Research over the past two decades has shown that inflammation in WAT is a major contributing factor in the hypermetabolic response observed in burn patients. WAT acts as an endocrine organ and plays an active role in secreting inflammatory moieties such as cytokines, hormones, and other growth factors. Under critical stress, adipocytes recruit inflammatory mediators, chemo-attractants (such as monocyte chemoattractant protein-1 or MCP-1) and cytokines that activate macrophage polarization as well as multiple metabolic signaling pathways. It is established that burn injury results in structural, functional, and morphological changes in WAT, with enhanced levels of circulating WAT-derived adipokines, inflammatory mediators and hormones that are known to regulate WAT inflammation and metabolism (Jeschke et al., 2011). Studies in human patients and rodents have shown that neutralization of TNFα accelerates wound healing (Ashcroft et al., 2012). Assessment of WAT collected from burn patients revealed the enhanced leukocyte infiltration, macrophages, and activation of Nod-like inflammasome receptor-3 (NLRP3) protein that plays a crucial role in multiple signaling pathways (Stanojcic et al., 2014). Furthermore, studies elucidating the role of NLRP3 in WAT after burn injury shows that NLRP3 has an anti-browning effect and that genetic deletion of this inflammasome augments WAT browning and the hypermetabolic response (Vinaik et al., 2019). Macrophage recruitment in WAT, on the other hand, undergoes alternate polarization and activation leading to the secretion of catecholamines and cytokines which induce multiple signaling cascades (Abdullahi et al., 2019a). For example, alternatively activated macrophages secrete IL6 that plays a crucial role in activating WAT browning and associated dysfunction post-burn injury (Ashcroft et al., 2012). Moreover, inhibition of alternatively activated macrophages impairs metabolic adaptation and the thermogenic response of adipose tissue. Furthermore, administration of interleukin-4 reinstates thermogenic gene expression, systemic fat mobilization and energy homeostasis in response to cold (Nguyen et al., 2011), suggesting the important role of alternatively activated macrophages in orchestrating the thermogenic response and metabolic adaptation of adipose tissue in response to stressful stimuli such as cold or burn injury.

## Therapeutic Advances Targeting WAT Lipolysis and Browning

Therapeutic interventions targeting β-adrenergic receptors, cytokines, WAT lipolysis, and browning mediators after burn injury have shown promising results in improving REE, hepatic steatosis, reducing hyperglycemia, hyperlipidemia, and chronic inflammation. Propranolol, a non-selective β-adrenergic signaling blocker, has shown promising benefits in reducing REE and reduction in the expression of browning markers (*Ucp1*, *Cox-iv*) in WAT, suggesting the importance of regulating catecholamines and stress hormones to mitigate WAT-associated dysfunction (Sidossis et al., 2015). Propranolol has higher affinity toward β1 and β2 receptors (Barbe et al., 1996; Finnerty and Herndon, 2013). However, studies conducted by an independent research group has shown that chronic adrenergic stress post-burn injury upregulates β3 receptor expression in WAT albeit its role in WAT browning remains elusive (Saraf et al., 2015). Also, studies in pediatric burn patients treated with propranolol by an independent group has shown a decrease in cardiac workload accompanied by reduced lipolysis, muscle catabolism, hepatosteatosis, and ultimately REE (Finnerty and Herndon, 2013). However, the non-selective nature of propranolol also exposes patients to a significant risk of cardiac failure (Unpublished clinical data). Furthermore, systemic IL6 levels were found upregulated in burn patient samples soon after burn injury and persisted for more than a month (Patsouris et al., 2015). Subsequent studies assessing the role of IL6 in WAT using the global IL6 knockout mice model has shown that deletion of IL6 prevents WAT browning after burn injury (Abdullahi et al., 2017). Also, IL6 deletion reduces infiltration of macrophages and inhibits alternative activation and polarization of macrophages (Abdullahi et al., 2019a). Further studies assessing the role of IL6 receptor blockade using Tocilizumab have shown positive results in inhibiting IL6 signaling and attenuating WAT browning and hepatic steatosis in the C57BL/6 murine model after burn injury (Abdullahi et al., 2019b), suggesting the therapeutic potential of Tocilizumab in reducing the WAT-associated hypermetabolic response in burn patients having enhanced IL6 levels.

Metformin, a successful clinical drug for use against diabetes has shown promising results in improving insulin resistance without causing hypoglycemia in phase II randomized clinical trials conducted in burn patients (Jeschke et al., 2016). Mechanistic studies in a murine model of thermal injury assessing the action of metformin has demonstrated that metformin induces protein phosphatase 2A (PP2A) activity, thus dephosphorylating key enzymes in the WAT lipolytic pathway [such as acetyl-CoA carboxylase (ACC) and HSL] and promoting fat storage in adipocytes (Auger et al., 2019). In fact, metformin treatment also reduces mitochondrial respiration and enhances mitochondrial coupling control in WAT, suggesting an indirect protective effect of metformin in reducing WAT browning and REE (Auger et al., 2019). While these changes are independent of adenosine monophosphate kinase (AMPK) activation, the canonical mechanism of metformin, the authors postulate that higher concentrations of this biguanide would be necessary to activate this signaling pathway in highly energetic beige adipose (Auger et al., 2019). Indeed, after hepatic uptake, it has been demonstrated in animal models that only 10–40 μM of metformin is bioavailable systemically, which may be insufficient to trigger AMPK’s pro-catabolic effects as well as the increase in mitochondrial biogenesis via PGC-1α (He and Wondisford, 2015). Additionally, another clinical study assessing metformin has shown promising results against skeletal muscle catabolism and insulin resistance following severe burn injury (Gore et al., 2005). Furthermore, studies assessing the impact of metformin on inflammation in adipocytes has revealed that metformin administration suppresses pro-inflammatory cytokines such as TNFα and IL-1β and also, indirectly enhances the anti-inflammatory effect of metformin (Qi et al., 2017). Moreover, in burn patients there is evidence that this biguanide can decrease inflammatory mediators in the serum such as IL-1β and MCP-1 (Jeschke et al., 2016). To date, the potential benefits of other biguanides or PPARγ agonists such as thiazolidinediones on glucose control and systemic dysfunction post-burn have not been adequately explored.

Acipimox, a niacin derivative that targets WAT lipolysis, has shown effective results in a murine model when challenged with severe burn injury. Acipimox not only reduced systemic lipid levels, in fact, it also attenuated WAT browning and hepatic fat infiltration after burn injury (Barayan et al., 2019), suggesting the therapeutic potential of acipimox in combating the hypermetabolic response after burn injury is via mitigating WAT dysfunction and associated complications. Additionally, acipimox has shown promising results in 3-month clinical trials in HIV-infected patients (23) with hyperlipidemia, and abnormal fat distribution. Acipimox treatment resulted in reduced systemic lipid levels, decreased WAT lipolysis and enhanced insulin sensitivity in HIV-infected patients (Hadigan et al., 2006). However, the impact of acipimox on WAT inflammation and systemic glucose metabolism in these adverse events has yet to be elucidated.

## Summary and Conclusion

**FIGURE 2:**
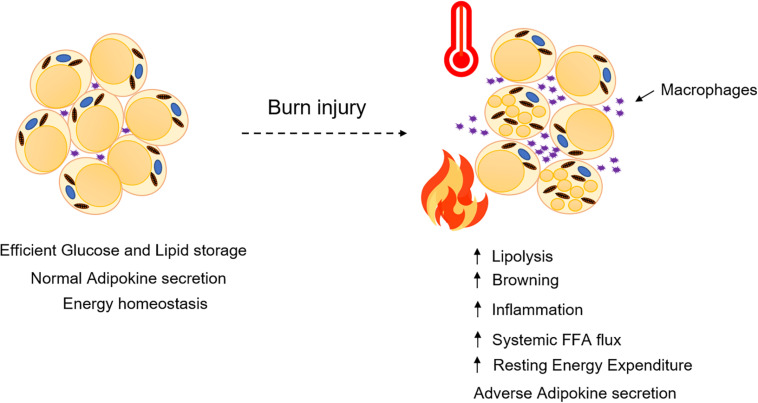
Adipose dysfunction and associated multi-organ damage after burn injury. Elevated levels of systemic FFA flux, inflammatory mediators and adipokines collectively contribute to a feed-forward loop, hypermetabolism, and multi-organ damage (Jeschke et al., 2020).

Adipose tissue has an enormous buffering capacity for release, storage, and dissipating energy in times of need. Research over recent years has made it clear that adipose tissue function and dysfunction has a major role to play in burn injury and its associated hypermetabolic response which often progresses to multi-organ dysfunction (Figure 2). Being an endocrine organ, the adipose tissue secretes a myriad of adipokines and maintains energy homeostasis in humans. Although significant success has been achieved in understanding the factors that trigger adipocyte dysfunction, our knowledge is still limited to the tip of an iceberg. Much of the research in the field has focused on identifying the key markers being altered when challenged by burn injury. Although the role of insulin has been thoroughly covered, there are a plethora of cytokines, adipokines (leptin, adiponectin) and stress hormones that are still not fully understood and how they affect insulin action and WAT morphology is still a matter of debate.

Macrophage infiltration and polarization in adipose tissue after burn injury is known, however, the exact role of inflammatory mediators is still not clear. Studies elucidating the role of IL6 and its inhibition have revealed the detrimental role of this cytokine in the processes of WAT browning and hepatic steatosis (Abdullahi et al., 2017, 2019b). However, its role in macrophage recruitment and polarization is not clear (Abdullahi et al., 2019a). Similarly, studies conducted to understand the role of TNFα in burn patients have revealed that it is upregulated after burn injury (Yeh et al., 1997), however, its exact role is still not clear. Research studies conducted in the NLRP3 murine model have demonstrated that deleting NLRP3 augments WAT browning, lipolysis, hepatic steatosis and impairs wound healing (Stanojcic et al., 2014; Vinaik et al., 2018, 2019), suggesting that inflammatory mediators have beneficial effects in the acute phase post-burn. However, further research studies are required to understand the protective role and mechanistic action of NLRP3 when challenged with burn injury.

**FIGURE 3:**
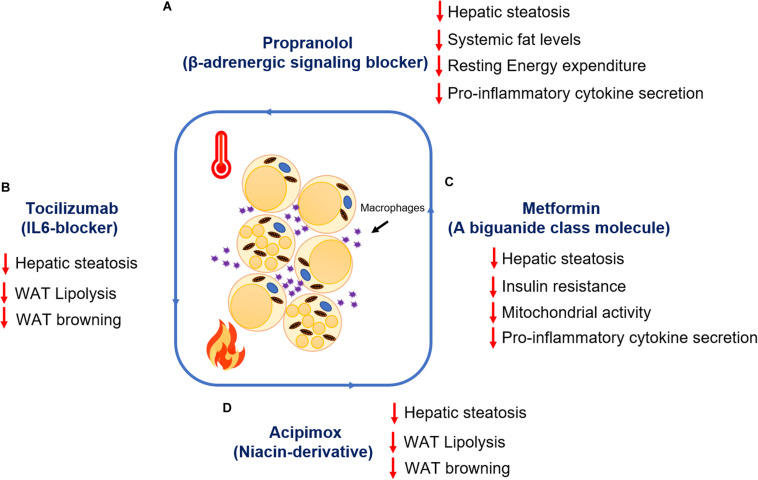
Summary of the therapeutic advances targeting adipocyte lipolysis and browning post-burn injury. Metabolic impact of drug treatment **(A)** Propranolol **(B)** Tocilizumab **(C)** Metformin **(D)** Acipimox post-burn injury.

Research over the past decade and advances in clinical burn care have significantly advanced our knowledge and greatly improved the survival of burn patients (Figure 3). Interventions such as metformin have shown promising safety and efficacy in phase II clinical trials. Moreover, metformin protects against WAT lipolysis, browning, and helps in maintaining euglycemia. Molecular studies of metformin’s mechanism of action have demonstrated the pathways activated to mitigate WAT dysfunction in rodent studies. Further detailed clinical investigation is, however, required to elucidate its effect on adipose tissue function after severe burn injury. Recently, the WAT lipolysis inhibitor acipimox has shown promising results in rodent studies when challenged with burn injury, suggesting the possible benefits of targeting WAT dysfunction in the future. However, further mechanistic studies are required to elucidate the action of acipimox and its possible impact on insulin sensitivity and WAT inflammation. The strong correlation of these drugs targeting WAT dysfunction suggests that reducing WAT lipolysis and browning is an important therapeutic strategy for the treatment of the hypermetabolic response in burn patients. Other potentially relevant strategies could be understanding the role of macrophage recruitment in WAT and mechanisms involved in activation and polarization of macrophages. To that effect, much remains to be uncovered with regards to the interactions of macrophages with themselves and the interaction of macrophages with adipocytes when challenged with burn injury. Lastly, understanding the role of adipokines and their impact on signaling pathways in vital target organs such as the brain, central nervous system, heart, liver, and skeletal muscle, can possibly reveal novel therapeutic strategies in reducing the WAT-associated hypermetabolic response in burn patients.

## Author Contributions

All authors listed have made a substantial, direct and intellectual contribution to the work, and approved it for publication.

## Conflict of Interest

The authors declare that the research was conducted in the absence of any commercial or financial relationships that could be construed as a potential conflict of interest.
